# *C* *lostridium difficile* surface proteins are anchored to the cell wall using CWB2 motifs that recognise the anionic polymer PSII

**DOI:** 10.1111/mmi.12958

**Published:** 2015-03-06

**Authors:** Stephanie E. Willing, Thomas Candela, Helen Alexandra Shaw, Zoe Seager, Stéphane Mesnage, Robert P. Fagan, Neil F. Fairweather

**Affiliations:** ^1^ Centre for Molecular Bacteriology and Infection, Department of Life SciencesImperial College LondonLondonSW7 2AZUK; ^2^ EA 4043, Universite Paris SudChâtenay‐MalabryFrance; ^3^ Krebs Institute, Department of Molecular Biology and BiotechnologyUniversity of SheffieldSheffieldS10 2TNUK

## Abstract

Gram‐positive surface proteins can be covalently or non‐covalently anchored to the cell wall and can impart important properties on the bacterium in respect of cell envelope organisation and interaction with the environment. We describe here a mechanism of protein anchoring involving tandem CWB2 motifs found in a large number of cell wall proteins in the Firmicutes. In the *Clostridium difficile* cell wall protein family, we show the three tandem repeats of the CWB2 motif are essential for correct anchoring to the cell wall. CWB2 repeats are non‐identical and cannot substitute for each other, as shown by the secretion into the culture supernatant of proteins containing variations in the patterns of repeats. A conserved Ile Leu Leu sequence within the CWB2 repeats is essential for correct anchoring, although a preceding proline residue is dispensable. We propose a likely genetic locus encoding synthesis of the anionic polymer PSII and, using RNA knock‐down of key genes, reveal subtle effects on cell wall composition. We show that the anionic polymer PSII binds two cell wall proteins, SlpA and Cwp2, and these interactions require the CWB2 repeats, defining a new mechanism of protein anchoring in Gram‐positive bacteria.

## Introduction

In order to colonise their hosts, bacterial pathogens produce a number of surface‐localised factors that promote survival in selected niches, including molecules that interact with host cells and cellular products, for example to promote adhesion and to circumvent the immune system (Pizarro‐Cerda and Cossart, 2006). Bacteria have evolved numerous strategies for displaying proteins on their surface including production of pili and flagella and the incorporation of proteins into the outer membrane of Gram‐negative bacteria. In Gram‐positive bacteria one well‐characterised mechanism for protein anchoring is that mediated by sortase enzymes that catalyse the covalent attachment of proteins to the peptidoglycan (Mazmanian *et al*., 1999; Schneewind and Missiakas, 2012). Other mechanisms responsible for non‐covalent anchoring of proteins to the cell wall of Gram‐positive bacteria have been described. These include LysM domains that bind to N‐acetylglucosamine (GlcNAc) residues of the peptidoglycan (Mesnage *et al*., 2014), CWB1 motifs that bind choline residues in teichoic acids, for example in the major autolysin LytA *Streptococcus pneumonia* (Fernandez‐Tornero *et al*., 2001) and GW motifs in *Listeria* internalin B that bind lipoteichoic acids (Jonquieres *et al*., 1999). Another well‐studied mechanism of non‐covalent anchoring involves three tandem surface layer homology (SLH) motifs within the protein. In *Bacillus anthracis*, the SLH motifs mediate binding to a pyruvylated secondary cell wall polysaccharide (SCWP) covalently linked to the peptidoglycan (Mesnage *et al*., 2000). The SLH motifs fold into a pseudo‐trimeric three‐pronged structure that is thought to interact with the pyruvate to mediate anchoring (Kern *et al*., 2011).

The majority of Clostridial surface proteins, including the S‐layer proteins, have three tandem copies of a motif termed CWB2 [reviewed in (Fagan and Fairweather, 2014)]. Over 150 species harbour proteins with CWB2 motifs, including *Clostridium difficile*, *C. tetani*, *C. botulinum* and many *Peptostreptococcaceae* (http://pfam.xfam.org/family/PF04122). The CWB2 motif, like the SLH motif, is complex and is incompletely conserved. SLH motifs are ∼55 amino acids in length, whereas CWB2 motifs are longer, ∼100 residues. However, despite performing similar functions, the two motifs share no apparent sequence homology. The *C. difficile* cell wall proteins (CWPs) and the S‐layer have been implicated as colonisation factors, enabling the bacterium to survive and proliferate in the intestine (Calabi *et al*., 2002; Merrigan *et al*., 2013; Spigaglia *et al*., 2013). In *C. difficile* the family of 29 CWPs, including SlpA, the adhesin Cwp66 and the phase‐variable protein CwpV all contain three tandem CWB2 motifs (Emerson *et al*., 2009; Fagan and Fairweather, 2011). Other CWPs contain putative peptidoglycan‐modifying activities (e.g. glucosaminidases, glycohydrolases and transpeptidases) or protease activities, e.g. Cwp84 and Cwp13 (Fagan and Fairweather, 2011; Fagan *et al*., 2011). This family of proteins can be released from the cell using chaotropic agents such as guanidinium or urea, confirming their non‐covalent anchoring to the cell wall.

*Clostridium difficile* is a Gram‐positive spore‐forming pathogen and is a leading cause of infectious diarrhoea worldwide (Rupnik *et al*., 2009; Cairns *et al*., 2012). Although widely associated with disease in the elderly within healthcare settings, *C. difficile* infection (CDI) can also occur in younger adults and children. Infections are also seen in animal populations, particularly in cattle and pigs (Rupnik *et al*., 2008). CDI commonly occurs following antibiotic treatment that alters the homeostasis of the gut microbiota, allowing *C. difficile* to proliferate and cause disease (Seekatz and Young, 2014). Transmission of *C. difficile* is facilitated by its ability to differentiate into endospores, highly robust, dormant forms of the bacterium that are resistant to environmental insult and difficult to eradicate (Lawley *et al*., 2010; Deakin *et al*., 2012). Most strains of *C. difficile* produce two protein toxins, TcdA and TcdB, that are considered the major virulence factors causing tissue damage leading to inflammation and tissue destruction (Voth and Ballard, 2005). Other factors necessary for successful colonisation and spread of infection are however less clear.

Recently three anionic polymers have been identified in *C. difficile*: PSI, PSII and PSIII (Ganeshapillai *et al*., 2008; Reid *et al*., 2012). PSI contains a penta‐glycosylphosphate repeating unit and is found in a minority of strains, whereas PSII and PSIII are found in all strains examined to date (including strain 630 used in this study). PSII consists of hexa‐glycosyl phosphate repeats (Ganeshapillai *et al*., 2008; Reid *et al*., 2012) and PSIII is a lipid‐bound glycosyl–phosphate polymer (Reid *et al*., 2012) and is described as a member of the extended lipoteichoic acid (LTA) family (Percy and Gründling, 2014). The roles of these anionic polymers in the lifestyle of *C. difficile* have not been studied, although their utility as antigens in vaccines has been explored (Bertolo *et al*., 2012; Cox *et al*., 2013).

Here we show that the CWB2 motifs mediate attachment of *C. difficile* CWPs to the cell wall and that the anionic polymer PSII serves as a ligand for this anchoring. We show that three CWB2 repeats are necessary for cell wall anchoring, and mutational analysis shows a role for a conserved amino acid sequence Ile Leu Leu (ILL) within the CWB2 motif.

## Results

### Three CWB2 motifs are required for anchoring of cell wall proteins

**Figure 1:**
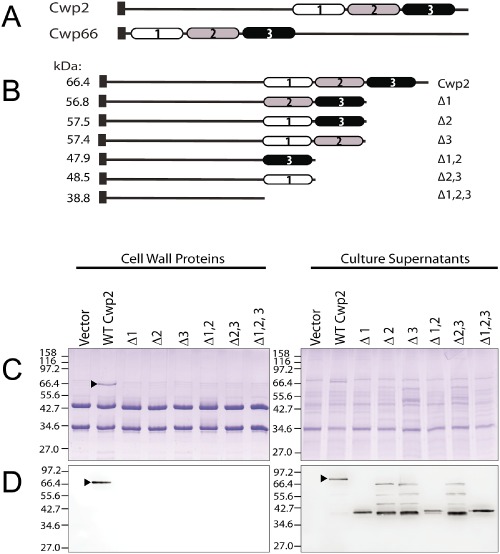
Deletion of the CWB2 repeats in Cwp2 prevents cell wall attachment in *C* *. difficile*. A. Domain structure of Cwp2 and Cwp66 showing locations of the CWB2 motifs (shaded). B. Deletion derivatives of Cwp2 constructed. C. SDS‐PAGE of cell wall extracts and culture supernatants from a *C* *. difficile cwp2* mutant containing plasmids expressing the deletion derivatives of Cwp2. D. Western blots using anti‐Cwp2 antibody. The numbering of the non‐identical CWB2 motifs is indicated. ▶, full length Cwp2. Molecular weights expressed in kDa.

In our previous studies, we observed that the three CWB2 motifs present in the cell wall protein CwpV were necessary for the anchoring of the protein to the cell (Reynolds *et al*., 2011). To investigate in detail the role of the CWB2 motifs in anchoring, we studied two cell wall proteins of *C. difficile*: Cwp2, a highly conserved surface protein, and Cwp66, a putative adhesin (Waligora *et al*., 2001). Both proteins are ∼66 kDa in size. In the CWP protein family the three CWB2 motifs can be located at either the N‐terminal or C‐terminal end of the protein, for example the motifs are C‐terminal in Cwp2 but N‐terminal in Cwp66 (Fig. 1A). The CWB2 motifs within Cwp2 were systematically deleted (Fig. 1B) and plasmids introduced into *C. difficile cwp2*::*erm*, a *cwp2* insertional mutant. A band of 66 kDa is visible in the S‐layer extracts of the *cwp2* mutant expressing full length Cwp2; this band is absent in the *cwp2* mutant containing the vector only (Fig. 1C). However, cell wall extracts from strains expressing any variant of Cwp2 containing CWB2 motif deletions (Δ1, Δ2, Δ3, Δ1,2, Δ2,3 and Δ1,2,3) appear similar to the *cwp2* mutant containing the control vector, suggesting these proteins are not maintained on the cell wall. Western blotting of these S‐layer extracts with anti‐Cwp2 antisera confirmed that only WT Cwp2 was anchored to the cell wall (Fig. 1D). In the culture supernatants, an immunoreactive band of 66 kDa is visible in the *cwp2* mutant expressing full length Cwp2 (Fig. 1D) but not in the mutant strain containing the empty vector. The strains containing the various CWB2 motif deletions of Cwp2 exhibited a pattern of degradation in the culture supernatants, which presumably results from inherent protein instability or the action of proteases. This result demonstrates that the proteins are expressed and secreted rather than anchored to the cell wall. To ensure the effects observed were not a specific feature of Cwp2, the experiment was repeated using the cell wall protein Cwp66 in a *cwp66* mutant background. Similar results were obtained, with deletion of one, two or three CWB2 motifs causing loss of cell wall anchoring, secretion of the proteins into the culture supernatant and protein degradation (Fig. S1).

### ILL sequences within CWB2 motifs are required for anchoring

**Figure 2:**
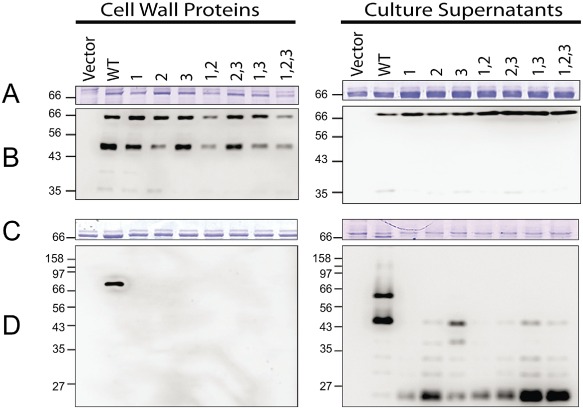
Effects of mutation of the PILL motif within CWB2 motifs. Derivatives of Cwp66 were constructed containing Pro to Ala or PILL to AAAA substitutions in one or more of the three CWB2 motifs as indicated and expressed in a *cwp66* mutant background. A and B. Pro to Ala substitutions. C and D, PILL to AAAA substitutions. Cell wall extracts (left) and culture supernatants (right) were analysed by SDS‐PAGE (A and C) and by Western blotting using anti‐Cwp66 antibody (B and D). Molecular weights expressed in kDa.

Although overall sequence conservation within and between CWB2 motifs present in *C. difficile* CWPs is low overall (average sequence identity between motifs is 27%), there are regions of high sequence conservation. The amino acid sequence Pro, Ile/Leu/Val, Ile/Leu/Val, Ile/Val (‘PILL’) is highly conserved in the 29 proteins of the *C. difficile* CWP family, with all three CWB2 motifs in most CWPs containing PILL, including SlpA, Cwp2, Cwp66 and CwpV (Fig. S2). As the proline residue was highly conserved within the PILL motifs, it was mutated to alanine in one, two or all of the repeats of Cwp66 and expressed in the *cwp66* mutant. Coomassie‐stained SDS‐PAGE and anti‐Cwp66 Western blotting of cell wall extracts and culture supernatant preparations (Fig. 2A and B) demonstrated that all proteins were localised in the cell wall. The levels of the mutated Cwp66 proteins in the cell wall extract are comparable with that of wild‐type Cwp66, suggesting that loss of the proline residue(s) did not compromise the anchoring ability of the proteins. This was confirmed by comparable levels of the wild type and mutated Cwp66 proteins being shed into the culture supernatant.

In order to determine the importance of the entire PILL motif in cell wall protein anchoring, the PILL residues were mutated *en bloc* to AAAA in either one, two or all three of the CWB2 repeats of Cwp66. The proteins were then expressed in the *cwp66* mutant and the effect on cell wall protein anchoring analysed by SDS‐PAGE and Western blotting (Fig. 2C and D). Only wild‐type Cwp66 was found in the cell wall extracts. Mutant Cwp66 proteins where one or more CWB2 motifs contained a PILL to AAAA substitution were not anchored to the cell wall but were instead found degraded in the culture supernatant (Fig. 2D).

### The relative order of CWB2 motifs is important for correct cell wall anchoring

**Figure 3:**
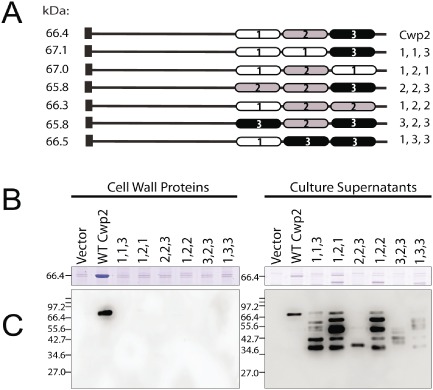
The relative order of CWB2 repeats is important for cell wall anchoring. The order of the CWB2 repeats was altered in a series of plasmids and expression carried out in a *cwp2* mutant background. A. SDS‐PAGE of Cwp2 proteins containing altered order of CWB2 repeats. B. Cell wall proteins and supernatants were analysed by Coomassie blue staining. C. Western blotting using anti‐Cwp2 antibody. Molecular weights expressed in kDa.

The three tandem copies of the CWB2 motif are necessary for anchoring (Fig. 1). However, because the motifs are not identical, we wondered whether the relative order of the motifs was important. A series of variants of Cwp2 was therefore constructed where one CWB2 motif was substituted one another. For instance, repeat 2 was removed and replaced with repeat 1, so that Cwp2 contained repeats 1, 1 and 3 in that order. The variant proteins generated are illustrated in Fig. 3A. These proteins were expressed constitutively in the *cwp2* mutant and cell wall extracts and culture supernatants were analysed. As expected a band at 66 kDa in the cell wall extract can be seen when wild‐type Cwp2 is expressed (Fig. 3B). In all cases where a Cwp2 variant is expressed, this 66 kDa band is not visible (Fig. 3B). Western blotting confirms lack of cell wall anchoring of the variants, but their presence in the culture supernatants as a series of lower molecular weight species confirms their expression and shows that they undergo considerable degradation (Fig. 3C).

### Identification of a locus required for CWB2‐mediated cell surface anchoring

**Figure 4:**
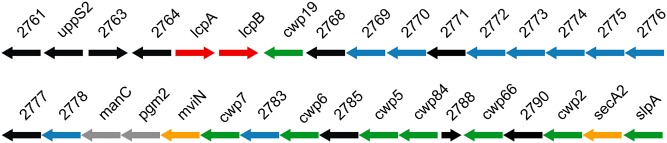
Genetic locus encoding the major cell wall proteins and anionic polymers of *C* *. difficile* 630. Gene names and colours indicate putative functions: green, cell wall or S‐layer proteins; blue, glycosyltransferases; red, attachment of polymers to peptidoglycan; grey, mannose biosynthesis; orange, protein translocation; black, other or unknown functions. Genes 2768–2778 are homologous to glycosyltransferases including those involved in teichoic and teichuronic acid biosynthesis in other Firmicutes.

Non‐covalent cell wall anchoring of surface proteins in Gram‐positive bacteria is often mediated via interaction with SCWPs, for example the S‐layer proteins Sap and EA1 of *B. anthracis* bind to pyruvylated SCWPs (Mesnage *et al*., 2000; Kern *et al*., 2010). We therefore analysed the genome for loci that may encode the synthesis of complex polysaccharides. We identified one locus within the *C. difficile* 630 genome encoding several glycosyltransferases and other proteins with homology to those involved in surface polymer biosynthesis in other Gram‐positive species. This locus, which we term the anionic polymer locus (AP locus), is located immediately downstream of the large S‐layer biogenesis locus encoding the S‐layer protein SlpA, the secretion ATPase SecA2 and the S‐layer maturation protease Cwp84 in addition to several other cell wall proteins including Cwp2 and Cwp66 (Calabi *et al*., 2001; Sebaihia *et al*., 2006) (Fig. 4). No genetic locus has been identified to encode the biosynthetic pathways for anionic polymers PSI, PSII and PSIII, and we considered that the AP locus could direct the synthesis of one or more of these polymers. Of note, within the AP locus are genes encoding a predicted phosphomannomutase (*pgm2*), which converts mannose‐1‐phosphate to mannose‐6‐phosphate, a predicted mannose‐1‐phosphate guanylyltransferase (*manC*), which is involved in the biosynthesis of GDP‐mannose and a second copy of a putative undecaprenyl pyrophosphate (UndPP) synthase (*uppS2*), which directs synthesis of UndPP, a precursor of undecprenyl phosphate (UndP) that serves as a lipid carrier for SCWP biosynthesis (reviewed in (Whitfield, 2006). Also present is CD2781, a homologue of the *E. coli* and *Salmonella mviN* (*murJ*) gene, a putative flippase that is proposed to mediate transfer of lipid II to the external surface of the membrane (Sham *et al*., 2014). As the PSII polymer contains mannose, and no other locus contains both these putative mannose biosynthetic genes, it is likely the AP locus directs synthesis of at least the PSII polymer.

**Figure 5:**
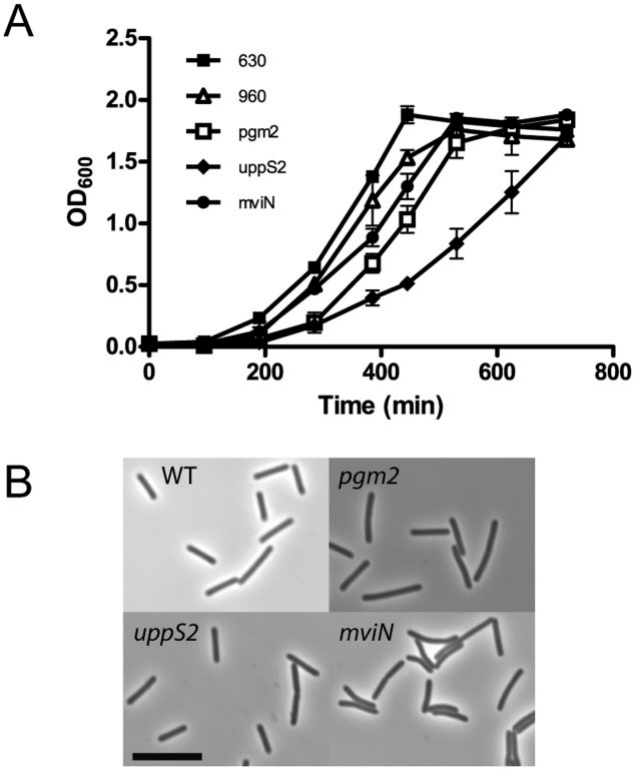
RNA knock‐down of genes within AP locus leads to growth defects. Strains were diluted from overnight cultures into BHIS broth containing 500 ng ml^−1^ anhydrotetracycline to induce anti‐sense RNA expression. A. Growth curve. B. Phase contrast images of representative bacteria from each culture. Scale bar = 10 μM.

Attempts to use a group II intron to insertionally inactivate CD2780 (*pgm2*), CD2775 (CDP‐glycerol:polyglycerol phosphate glycerophosphotransferase) and CD2762 (*uppS2*) consistently failed, suggesting these genes may be essential for bacterial growth (data not shown). We decided to use inducible anti‐sense RNA to knock‐down expression as described previously (Fagan and Fairweather, 2011). Three plasmids were constructed, which contained antisense targeted to CD2780 (*pgm2*) (pSEW036), CD2762 (*uppS2*) (pSEW037) and CD2781 (pSEW038). The plasmids contain ∼ 170 bp of DNA antisense to the 5′ of the target mRNA, including the translational start site and ribosome binding site under the control of a tetracycline‐inducible promoter. To assess the effects of expression of the antisense RNA on cell growth, cultures of *C. difficile* 630 containing pSEW036, pSEW037 or pSEW038 were grown overnight in Brain Heart Infusion Supplemented (BHIS) broth without the inducer anhydrotetracycline and sub‐cultured in the same medium containing anhydrotetracycline (500 ng ml^−1^). Slight growth defects were seen for cultures containing pSEW036 and pSEW038, which express antisense to *pgm* and *mviN*, respectively, and a severe growth defect was seen for pSEW037, which expresses antisense to *uppS2* (Fig. 5A). Successful depletion of the target mRNA in all three cultures was confirmed by semi‐quantitative reverse transcription PCR (Fig. S3). Phase contrast microscopy revealed an increase in overall cell length for strains expressing anti‐sense to *pgm2* (5.31 μm ± 2.03) and *uppS2* (5.28 μm ± 2.21) compared with 630 carrying a control plasmid (4.4 μm ± 1.13) and slight curvature to some cells expressing anti‐sense to *mviN* (Fig. 5B).

**Figure 6:**
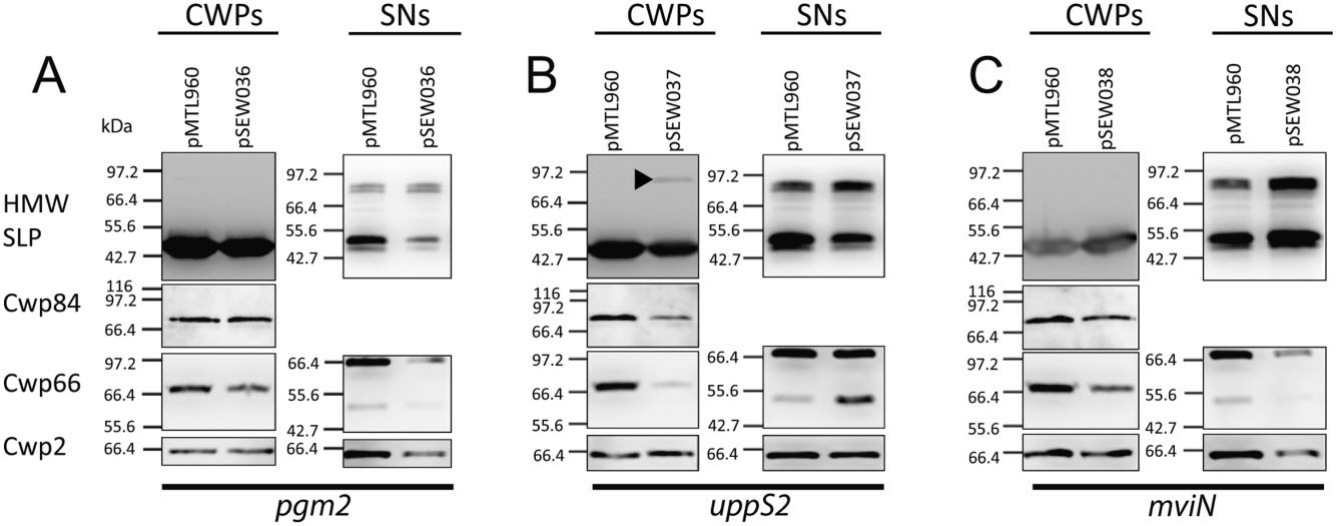
The cell envelope is compromised in strains expressing RNA antisense to *pgm*, *uppS* and *mviN*. Strains were diluted from overnight cultures into BHIS broth containing 500 ng ml^−1^ anhydrotetracycline to induce anti‐sense RNA expression. After 6 h, cell wall extracts and culture supernatants were prepared and Western blotted using antibodies as indicated. In the culture expressing anti‐sense to *uppS*, the band indicated with ▶ represents full length SlpA. pMTL960, vector control; pSEW036, *pgm* antisense; pSEW037, *uppS* antisense; pSEW038, *mviN* antisense. Samples were standardised based on the OD_600_ of the culture. Molecular weights expressed in kDa.

To investigate the effects of RNA knock‐down on formation of the cell wall, extracts and culture supernatants were analysed by SDS‐PAGE and Western blotting. In all three strains the levels of Cwp66 in the cell wall appeared to be reduced, implying knock‐down of the three targeted genes affects S‐layer assembly (Fig. 6). Further aberrations in S‐layer assembly were apparent in strain 630 (pSEW037), expressing RNA antisense to *uppS2*. Unlike Cwp2 and Cwp66, SlpA is efficiently post‐translationally cleaved by a protease, Cwp84, into two subunits: the HMW and LMW SLPs (Kirby *et al*., 2009). The presence of full‐length SlpA on the cell wall has only been previously reported in a *cwp84* mutant, where the protease responsible for cleaving SlpA into the HMW and LMW SLPs has been inactivated (Kirby *et al*., 2009). Therefore, the presence of full‐length SlpA on the cell wall is highly suggestive of disrupted S‐layer assembly. We observed full‐length unprocessed SlpA in the cell wall of strain 630 containing plasmid pSEW037, as seen by Western blotting with anti‐HMW (Fig. 6). Immunoblotting with anti‐Cwp84 antibody revealed less Cwp84 in the cell wall of 630 (pSEW037) consistent with the appearance of unprocessed SlpA. In contrast to a *cwp84* mutant, the appearance of full‐length SlpA and loss of Cwp66 on the cell wall upon expression of *uppS2* anti‐sense RNA did not appear to coincide with a large increase of these proteins in the culture supernatant. Together these experiments suggest that genes downstream of the major cell wall protein locus encode synthesis of glycan polymers that are involved in the integrity of the cell envelope.

### Tunicamycin does not inhibit CWB2‐mediated wall anchoring

**Figure 7:**
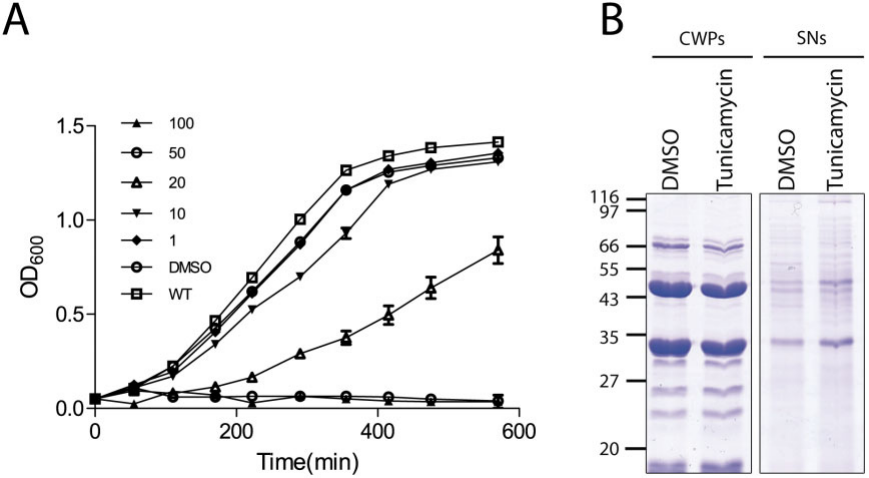
Effect of tumicamycin on the cell wall *C* *. difficile 630*. Cells were grown in BHIS broth supplemented with 20 μg ml^−1^ tunicamycin to an OD of 0.85 before extracting cell wall proteins with low pH glycine and precipitating the proteins in the culture supernatant. A. Growth curve in the presence of varying concentrations (μg/ml) of tunicamycin as indicated or the solvent DMSO. B. Cell wall proteins (CWPs) and culture supernatant proteins (SNs) from cells grown in 20 μg ml^−1^ tunicamycin. Molecular weights expressed in kDa.

A homologue of *tagO* encodes the initiating glycosyltransferase of all secondary cell wall polymer biosynthesis pathways studied to date, with the exception of *S. pneumoniae*, where the sugar transferase Spr1655 is predicted to attach the unusual sugar AATGal to UndP (Denapaite *et al*., 2012). Along with TagA, TagO is involved in the biosynthesis of the GlcNAc‐ManNAc linkage unit that links the repeating unit of the SCWP to the peptidoglycan. In *B. subtilis* and *S. aureus*, genes encoding proteins involved in the biosynthesis of teichoic acids are essential unless one of the first two genes in the pathway, *tagO* or *tagA*, is inactivated, presumably due to the sequestration of the lipid carrier undecaprenyl phosphate. Identifying the initiating glycosyltransferase is therefore of interest. BLAST searches of the *C. difficile* 630 genome failed to identify a TagO homolog (data not shown), but the possibility remained that *C. difficile* contains a functional *tagO* homologue. To investigate this, we used the antibiotic tunicamycin, a potent and highly selective inhibitor of TagO; it can also inhibit MraY, but its affinity for MraY compared with TagO is at least 100‐fold lower (Swoboda *et al*., 2010). In *S. aureus*, a reduction in wall teichoic acid (WTA) content is observed when cells are treated when tunicamycin at 0.02 μg ml^−1^, and WTA is depleted at 0.20 μg ml^−1^; an associated growth defect relative to untreated cells is also apparent (Campbell *et al*., 2011). Growing *C. difficile* in similar low concentrations of tunicamycin did not lead to any growth defects (data not shown); therefore, higher concentrations of tunicamycin were used. A growth defect relative to the dimethyl sulphoxide‐treated cultures was observed at 10 μg ml^−1^; at the two highest concentrations used (50 μg ml^−1^ and 100 μg ml^−1^) no growth occurred (Fig. 7A). If tunicamycin was inhibiting an enzyme involved in anionic cell wall polymer biosynthesis in *C. difficile*, we might expect a change in the cell wall proteins profile. Cells were grown in BHIS broth containing 20 μg ml^−1^ tunicamycin, and cell wall proteins in the culture supernatants were prepared. The profile of the cell wall proteins from cells grown in the presence of tunicamycin was similar to untreated cells (Fig. 7B). However, there appeared to be more shedding of proteins into the culture supernatant in the tunicamycin treated cells, but it is likely this is due to cell stress and/or inhibition of MraY.

### The anionic polymer PSII is the ligand recognised by CWB2 domains

**Figure 8:**
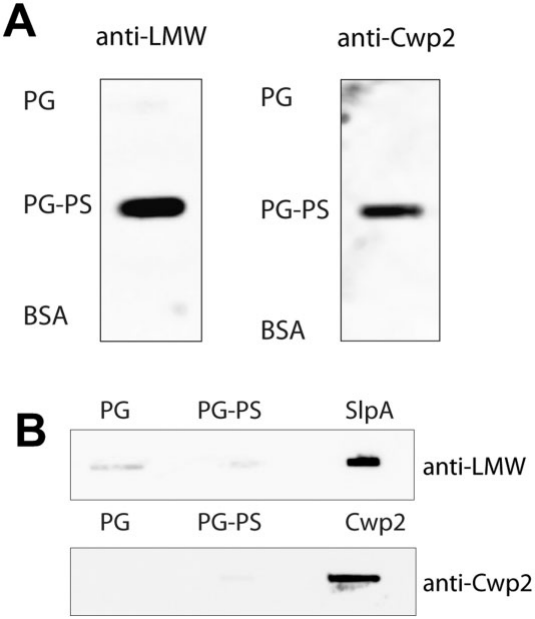
A. Binding of SlpA and Cwp2 to the polysaccharide fraction of a peptidoglycan–polysaccharide complex. Ten micrograms of peptidoglycan (PG; prepared by HF‐treatment of PG‐PS), peptidoglycan‐polysaccharide (PG‐PS) or bovine serum albumin (BSA) were spotted on to a PVDF membrane. The membrane was then incubated with 25 μg ml^−1^ of protein (SlpA‐His or Cwp2‐His) for 45 min. The membrane was washed three times with PBS before blotting with anti‐LMW for SlpA detection or anti‐Cwp2 for Cwp2 detection, followed by an HRP‐conjugated secondary antibody. SlpA and Cwp2 bind to PG‐PS but not to PG. B. Control dot‐blots showing antibodies to LMW SLP (SlpA) and Cwp2 do not react with PG or PG‐PS. Samples of PG, PG‐PS or protein were applied to the filter and reacted with anti‐Cwp2 or anti‐LMW SLP antibodies.

In other Gram‐positive species, S‐layer proteins have been shown to be anchored to the cell wall via non‐covalent interactions with secondary cell wall polysaccharides (see above). Given that the AP locus downstream of the major CWP genes likely encodes for PSII, we next investigated whether the ubiquitous anionic polymer PSII, present in all strains including 630 used in this study, has a role in anchoring CWPs to the cell wall. Peptidoglycan and covalently bound polymers (PG‐PS) was isolated from *C. difficile* 630 and the polymers released by hydrofluoric acid (HF) treatment to generate free peptidoglycan (PG) as described previously (Candela *et al*., 2011). To test for their ability to bind SlpA and Cwp2, PG and PG‐PS were immobilised on a polyvinylidene fluoride (PVDF) membrane followed by incubation with purified SlpA or Cwp2 protein. Binding of protein was detected by immunoblotting. As shown in Fig. 8A, both proteins bound PG‐PS and not to PG, indicating that a component attached to the peptidoglycan, and not peptidoglycan alone, is a ligand for these proteins. Control experiments showed that the anti‐LMW SLP and anti‐Cwp2 antibodies do not react with PG‐PS or PG (Fig. 8B).

**Figure 9:**
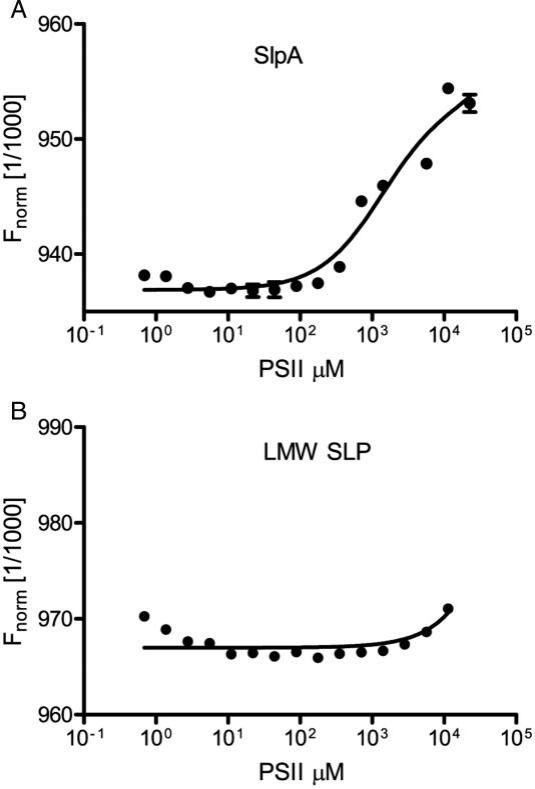
Binding of PSII polysaccharide to LMW SLP. PSII polysaccharide was extracted from *C* *. difficile* using acetic acid and titrated against 50 mM full‐length SlpA (A) or LMW SLP (B) using microscale thermophoresis. Binding was detected with only with SlpA, which contains 3 × CWB2 motifs. NMR analysis of the polysaccharide confirmed a similar structure to PSII (Fig. S2).

The interaction between SlpA and the peptidoglycan‐attached anionic polymer was studied further using microscale thermophoresis. For these experiments, the PS fraction was recovered from purified cell walls by acetic acid hydrolysis, a treatment that releases the PSII polysaccharide (Ganeshapillai *et al*., 2008). Acetic acid is a gentler treatment than HF and is expected to preserve a greater degree of structural integrity within the polymer, which may be functionally important. The purity of the released polymer was confirmed by nuclear magnetic resonance analysis (Fig. S4). LMW SLP (the N‐terminal portion of SlpA following cleavage by Cwp84) was used as a negative control as it does not contain any CWB2 motifs. PSII was titrated against 50 nM His‐tagged LMW SLP or His‐tagged SlpA fluorescently labelled with NT‐647‐NHS. As expected, LMW SLP, which does not contain a CWB2 domain, did not interact with PSII (Fig. 9A) and revealed only a limited increase of signal at the lowest and highest concentration of PSII within an expected background noise range. In contrast, specific binding was detected when PSII was titrated against SlpA with an apparent affinity in the millimolar range. Due to the relatively low affinity of SlpA for PSII, a complete titration experiment could not be carried out, but the apparent affinity was estimated to be 1.5 mM ± 133 μM.

## Discussion

There are several well‐studied mechanisms of cell wall anchoring involving repeating motifs within surface proteins. Some of these mechanisms, like that of the LysM repeats, are additive with the repeats acting like ‘beads on a string’ and the strength of the interaction correlating to the number of repeats (Mesnage *et al*., 2014). Conversely SLH repeats, which occur predominately in triplicate within the protein, act together to form a tertiary structure necessary for binding (Kern *et al*., 2011) with the consequence that deletion of one SLH repeat abrogates cell wall anchoring. In our study, we investigated the CWB2 motif, found in triplicate at the N or C terminus of the 29 proteins that comprise the *C. difficile* CWP family and throughout many surface proteins of other Gram‐positive bacterial species.

Deletion of any of the three CWB2 motifs within either Cwp2 or Cwp66 protein lead to a complete loss of detectable cell wall anchoring, suggesting that the CWB2 motifs do not operate by a ‘beads on a string’ mechanism, but perhaps mediate binding by formation of a structure involving all three repeats, akin to the SLH repeats. This hypothesis is further reinforced by mutation of a conserved set of amino acid residues, P[ILV][ILV][LV], to AAAA in any single CWB2 repeat leading to a loss of CWP anchoring. Furthermore, if binding of the repeats was additive, we might expect the three CWB2 repeats within a given protein to be functionally equivalent. However, when we replaced one CWB2 repeat of Cwp2 with a different CWB2 repeat from the same protein, the protein was no longer anchored to the cell wall. Analysis of the amino acid sequences of the three CWB2 repeats found in the *C. difficile* CWPs reveals conserved differences between the repeats; for instance, repeat ‘3’ is consistently the shortest of the three repeats (data not shown). Collectively, these data suggest an evolutionary divergence of the repetitive CWB2 motifs.

To investigate possible ligands for the CWB2 motifs, we took advantage of the characterised anionic polysaccharides found in *C. difficile*. Peptidoglycan extracts containing the anionic polysaccharide PSII retained binding activity to both Cwp2 and SlpA, but hydrofluoric acid‐treated peptidoglycan, lacking polysaccharides, lost this activity, demonstrating that the CWB2 repeats bind to a SCWP rather than to the peptidoglycan itself. PSII appears to be highly conserved in *C. difficile* (Ganeshapillai *et al*., 2008; Reid *et al*., 2012). Using SlpA purified from *C. difficile*, an interaction with PSII was observed using microscale thermophoresis, and this was dependent on the presence of the CWB2 repeats. The PSII was from purified from *C. difficile* using acetic acid, thus avoiding complete breakage of the internal phosphodiester bonds within the polymer and potentially allowing PSII to retain more of its structural integrity. Thus, like the SLH repeats in *B. anthracis*, the CWB2 repeats also mediate non‐covalent anchoring to a SCWP.

We identified a likely AP locus for the biosynthesis of the anionic polymers PSII, located directly downstream of the major cell wall proteins. Insertional inactivation of genes in this locus was unsuccessful, suggesting these genes may be essential. Alternatively, such mutants could result in build up of toxic intermediates within the cell or the sequestration of limiting intermediates essential for other pathways, e.g. UndP. It is also possible that the introns did not successfully target genes in the AP pathway. However, there are data successfully data suggesting SCWP genes may be essential in other species. In *B. anthracis*, the SCWP may be essential for cell growth, as null mutants cannot be isolated in *tagO* or *tagA*, genes shown to be required for SCWP synthesis (Kern *et al*., 2010). However, our anti‐sense knock‐down of key genes led to a growth defect when expressed, most significantly with anti‐*uppS2*. This was accompanied by a reduction in the amount of Cwp66 anchored to the cell wall for all three of the targeted genes. A previous study of aberrations in cell wall protein anchoring due to inactivation of Cwp84, which processes the S‐layer, showed Cwp66 to be the most affected CWP, suggesting that this CWP is particularly sensitive to S‐layer perturbations (de la Riva *et al*., 2011). The clearest alteration to the profile of the cell wall proteins is observed when *uppS2* is targeted; unprocessed full‐length SlpA can be detected on the cell wall, and a reduced amount of Cwp84 is also apparent. The reduction in these CWPs on the cell wall does not appear to coincide with an increase in their presence in the culture supernatant. This contrasts to observations made in a *cwp84* mutant (de la Riva *et al*., 2011) where a decrease of a CWP on the cell wall correlated with an increase in that CWP in the culture supernatant. This hints at the possibility of a feedback mechanism linking levels of free SCWP on the cell wall and CWP secretion.

Our data demonstrate a link between the putative anionic polysaccharide biosynthesis locus and cell wall anchoring of surface proteins. Although it might be argued that the link is indirect and caused by general changes to the peptidoglycan structure rather than the CWB2 ligand specifically, we found the S‐layer profile unperturbed following treatment with high concentrations of tunicamycin, a known MraY and peptidoglycan biosynthesis inhibitor, suggesting that the cell wall proteins are not sufficiently sensitive to general peptidoglycan biosynthesis inhibition.

It is worth noting that *C. difficile* 630 encodes two *uppS* homologues, whereas BLAST searches indicate that the genomes *of E. coli* and *B. subtilis* encode only one UppS (data not shown). *C. difficile uppS*, encoded by CD2136, is located upstream of a phosphatidate cytidylyltransferase and downstream of a conserved hypothetical protein and a ribosome recycling factor. This protein shows higher homology to UppS of *B. subtilis*, (51.9 % amino acid sequence identity) than does UppS2 located in the putative AP locus (25.1 % amino acid sequence identity to *B. subtilis* UppS). This suggests CD21360 (*uppS1*) is a functional homologue of *uppS* that synthesises undecaprenyl UndPP for use in peptidoglycan biosynthesis, whereas CD27620 (*uppS2*) is potentially dedicated to the SCWP biosynthesis pathway in *C. difficile*, hinting at the importance of SCWPs in this organism. The AP locus is the only large polysaccharide biosynthetic locus in the *C. difficile* 630 genome aside from another (smaller) locus dedicated to flagellar glycosylation (Faulds‐Pain *et al*., 2014). The AP locus contains two genes involved in conversion of mannose‐6‐phospate to GDP‐mannose (CD2779 and 2780) and disruption of CD2780 gene *(pgm2)* expression by RNA knock‐down leads to loss of CWPs on the cell wall. Given PSII contains mannose, it is likely that the AP locus encodes for the biosynthesis of PSII. Further work is necessary to decipher the roles of individual genes within the AP locus and to determine the surface polysaccharides encoded. For example, all *C. difficile* strains produce PSII and the lipid anchored PSIII (LTA) and some also produce an additional anionic polymer PSI. Our data indicate the essentiality of some genes within the AP locus, which shows similarities to anionic polymer and teichoic acid biosynthetic loci from other Gram‐positive species. The SCWP of *B. anthracis* is similarly thought to be essential (Kern *et al*., 2010). However, it is also possible that, as in *B. subtilis* and *S. aureus*, the targeted genes are only conditionally essential in the presence of the initiating glycosyltransferase (D'Elia *et al*., 2006). That tunicamycin did not result in a substantial growth defect in our studies suggests that *C. difficile* does not produce a functional homologue of *tagO*, the initating glycosyltransferase found in *B. subtilis* and *S. aureus*. The presence of TagO in bacterial species appears to correlate with the presence of GlcNAc‐ManNAc linkage unit between the peptidoglycan and the repeating unit of the polymer [reviewed in (Brown *et al*., 2013)]. The lack of a TagO homologue in *C. difficile* suggests that PSII is not linked to peptidoglycan using this commonly found linkage, and perhaps does not contain any linkage unit.

In summary, our study provides insights into the mechanism of cell wall anchoring mediated by CWB2 motifs. These repeats are found in many species of Firmicutes, suggesting they reflect a widespread mechanism for anchoring of surface proteins within this phylum. Similarities are evident between the CWB2 and SLH motifs, including the requirement for three tandem motifs, and it is possible that structural characterisation of the CWB2 repeats will reveal them to form a pseudo‐trimer domain similar to that formed by the SLH repeats. The interaction between anionic polysaccharides and CWB2 motifs to facilitate surface protein anchoring uncovers an additional function for the plethora of activities recognised for surface polymers in Gram‐positive bacteria (Weidenmaier and Peschel, 2008).

## Experimental procedures

### Strains and plasmids

*Clostridium difficile* 630 (Sebaihia *et al*., 2006) and its erythromycin‐sensitive derivative 630Δ*erm* (Hussain *et al*., 2005) were grown under anaerobic conditions (80%N_2_, 10% CO_2_, 10% H_2_) in an anaerobic incubator (Don Whitley Scientific) on Brain Heart Infusion Supplemented (BHIS) plates or in broth [BHI supplemented with 5 g l^−1^ yeast extract (BD Bacto), and 1 g l^−1^ L‐cysteine]. *E. coli* strains were grown in LB broth and on LB agar supplemented with chloramphenicol (15 μg ml^−1^), kanamycin (50 μg ml^−1^) or carbenicillin (50 μg ml^−1^). *E. coli* strain NovaBlue (Merck) was used as a recipient for all recombinant plasmids, and strain CA434 (HB101 carrying R702) was used for conjugation of plasmids from *E. coli* into *C. difficile*. *E. coli* Rosetta carrying pLMW630 was described previously (Fagan *et al*., 2009).

### Genetic techniques

All plasmids used in this study are described in Table S1. Standard cloning procedures were used, and chromosomal DNA was purified from *C. difficile* as described (Emerson *et al*., 2009). Insertional mutagenesis of *C. difficile* 630Δ*erm* was carried out using the Clostron system (Heap *et al*., 2010). Retargeted vector plasmids based on pMTL007C‐E2 were designed using the intron design tool (http://www.clostron.com) and commercially synthesised (DNA 2.0) generating pHAS003 for targeting to *cwp2* and pZLS003 for targeting to *cwp66*. After conjugation from *E. coli* CA434 into *C. difficile* 630Δ*erm* and growth on Braziers CCEY agar containing cefoxitin, cycloserine and thiamphenicol to counter select against the *E. coli* donor. Erythromycin‐resistant colonies were screened by PCR for the presence of the chromosomally‐localised intron using the intron specific EBS primer NF1063 and gene‐specific primers (see Table S1).

Plasmids expressing Cwp2 or Cwp66 were constructed by amplifying the relevant coding sequence from strain 630, including terminal BamHI and SacII sites, and cloning into pRFP144, a derivative of pMTL960 containing the P*_cwp2_* promoter (Fagan and Fairweather, 2011) forming pHAS002 (for Cwp2) and pZLS002 (for Cwp66). Derivatives of pHAS002 and pZLS002 containing altered CWB2 repeats were constructed by PCR (Tables S1 and S2).

### RNA extraction, RNA knock‐downs and RT‐PCR

RNA was prepared as described previously (Fagan and Fairweather, 2011). RNA yield and quality were checked by spectrophotometry and by agarose gel electrophoresis. RNA was confirmed as DNA‐free by PCR using 16S rRNA primers (NF408 and NF409). RT‐PCR was performed using RETROscript First Strand Synthesis Kit (Ambion). Two microlitres of Random Decamers (50 μM) were added to 1 μg RNA to a total volume of 12 μl with nuclease free water, followed by 2 μl 10× RT buffer, 4 μl dNTPs (containing 2.5 mM each dNTP), 10 units RNase inhibitor and 100 units Moloney murine leukaemia virus reverse transcriptase and incubated at 44°C for 1 h. The enzyme was then heat‐inactivated at 92°C for 10 min. The reaction was not heated prior to the reverse transcription reaction to avoid disruption of antisense:mRNA duplexes. Two microlitres of generated cDNA was used as a template in a standard 20 μl Taq PCR reaction.

Expression of antisense RNA was carried out as described previously (Fagan and Fairweather, 2011). Using primers NF1810 + NF1811, NF1812 + NF1812 and NF1841 + NF1842, ∼170 bp of DNA antisense to the 5′ of the target mRNA, including the transcriptional start site and ribosome binding site was amplified and cloned into tetracycline‐inducible plasmid pRPF185, utilising the SacI and BamHI sites added to the primers, producing plasmids pSEW036, pSEW037 and pSEW038. Plasmids were transferred to *C. difficile* using thiamphenicol (15 μg ml^−1^) selection. Strains were grown overnight in BHIS and sub‐cultured the following morning to an OD_600_ of 0.025 in fresh BHIS supplemented with 15 μg ml^−1^ thiamphenicol and 500 ng ml^−1^ anhydrotetracycline.

### Preparation of cell extracts

Cell wall extracts were prepared as described previously (Fagan and Fairweather, 2010). Culture supernatants were prepared by addition of TCA to a final concentration of 10%, the samples vortexed briefly, and incubated on ice for 30 min. Samples were centrifuged at 15 000 *g* for 10 min at 4°C and the supernatant removed. The pellet was resuspended in 1 ml 90% pre‐chilled acetone and vortexed for 15 min. The 90% acetone wash step was repeated, and the final pellet was dried. SDS‐PAGE and Western immunoblotting were carried out using standard methods and as described previously using 12% polyacrylamide gels (de la Riva *et al*., 2011) using murine antibodies against Cwp2 (1 in 50 000), Cwp66 (1 in 20 000), Cwp84 (1 in 4000) and rabbit antibody against and LMW SLP (for SlpA; 1 in 200 000). Secondary antibodies conjugated to HRP (Dako) were applied (rabbit anti‐mouse 1 in 1000; goat anti‐rabbit 1 in 200) and the signal detected using SuperSignal West Pico chemiluminiscent substrate (Pierce).

### Preparation of *C* *. difficile* proteins

Cwp2‐His and SlpA‐His were expressed in *C. difficile* 630 containing pHAS005 and pSEW028 respectively. Five hundred millilitres of cultures were grown overnight in TYG broth; the cells were harvested and washed with 20 ml PBS and freeze‐thawed three times. Pellets were resuspended in 7 ml wash buffer (50 mM HEPES, 300 mM NaCl, 10 mM imidazole) containing 280 μg DNase I and 3.5 mg lysozyme and incubated at 37°C for 45 min. After centrifugation the supernatants were passed through a 0.2 μm filter and purified using an AKTA Prime with a 1 ml His‐Trap column (GE Healthcare). Proteins were eluted over a gradient of 0–250 mM imidazole in 50 mM HEPES, 300 mM NaCl. Proteins were then desalted into 10 mM HEPES 7.5, 150 mM NaCl using a HiTrap desalting column (GE Healthcare). His‐tagged LMW SLP protein was purified from *E. coli* Rosetta containing pLMW630 as described previously (Fagan *et al*., 2009). Protein concentrations were determined using BCA assay (Pierce) according to the manufacturer's instructions.

### Preparation of cell wall polymers

PG‐PS was purified as previously described (Candela *et al*., 2011). Cells were grown in 1 l TY medium to OD_600_ 1.0, pelleted and boiled for 10 min in 80 ml of 50 mM Tris‐HCl, pH7.4, 150 mM NaCl, 1% SDS. The suspension was centrifuged at 6500 *g* for 10 min and the boiling step repeated to obtain the peptidoglycan covalently linked to cell wall polysaccharide. The resulting pellet was washed in 40 ml of 50 mM Tris‐HCl, pH 7.4, sonicated and centrifuged at 50 000 *g* for 20 min. The remaining contaminants were discarded by treating the resulting pellet, solubilised in 20 mM MgSO_4_, by DNase (1 mg ml^−1^) and RNase (5 mg ml^−1^) for 2 h, then by proteinase K (20 μg ml^−1^ at room temperature for 12 h) in the presence of 10 mM CaCl_2_, and finally by SDS boiling (1% at 100°C for 10 min). Under these conditions, polysaccharides covalently linked to the peptidoglycan via a phosphodiester linkage co‐purify with the PG. To purify the PG, the PSII polysaccharide was released from the PG and degraded by incubation of the PG‐PS with hydrofluoric acid (48%; Sigma‐Aldrich) for 48 h at 4°C. PG was then washed three times in H_2_O and evaporated. To purify and analyse the PS covalently linked to the PG, the PS was partially released from the PG using 1% acetic acid (95°C, 1 h). After centrifugation, the supernatant was lyophilised and analysed by NMR (Brucker Advance 300). The sample was dissolved in D_2_O, and the spectra was recorded at 300K.

### Binding of proteins to cell wall components

Ten micrograms of PG and PG‐PS samples in a total volume of 10 μl were blotted on to a PVDF membrane (Pall) using a Minifold I Slot‐Blot 48‐well system (Whatman) attached to a vacuum pump. The membrane was incubated with 25 μg ml^−1^ of protein (Cwp2 or SlpA) at room temperature with agitation for 45 min. Membranes were washed three times with PBS before incubation with mouse anti‐Cwp2 (1 in 50 000) or rabbit anti‐LMW SLP (1 in 200 000) antibodies (1 h, room temperature). Secondary antibodies conjugated to HRP (Dako) were applied (rabbit anti‐mouse 1 in 1000; goat anti‐rabbit 1 in 2000) and the signal detected using SuperSignal West Pico chemiluminiscent substrate (Pierce).

## Supporting information

Supporting information
