# Supplementary material for: C lostridium difficile surface proteins are anchored to the cell wall using CWB2 motifs that recognise the anionic polymer PSII
Source: Mol Microbiol. 2015 Mar 6;96(3):596–608. doi: 10.1111/mmi.12958 (PMC4973711; doi:10.1111/mmi.12958)
Supplement: Supplementary file 1 — Supporting information [file MMI-96-596-s001.pdf]

Clostridium difficile surface proteins are anchored to the cell wall using CWB2 motifs that recognise the anionic polymer PSII

Stephanie E. Willing, Thomas Candela, Helen Alexandra Shaw, Zoe Seager, Stephane Mesnage, Robert P. Fagan and Neil F. Fairweather*

SUPPLEMENTARY INFORMATION

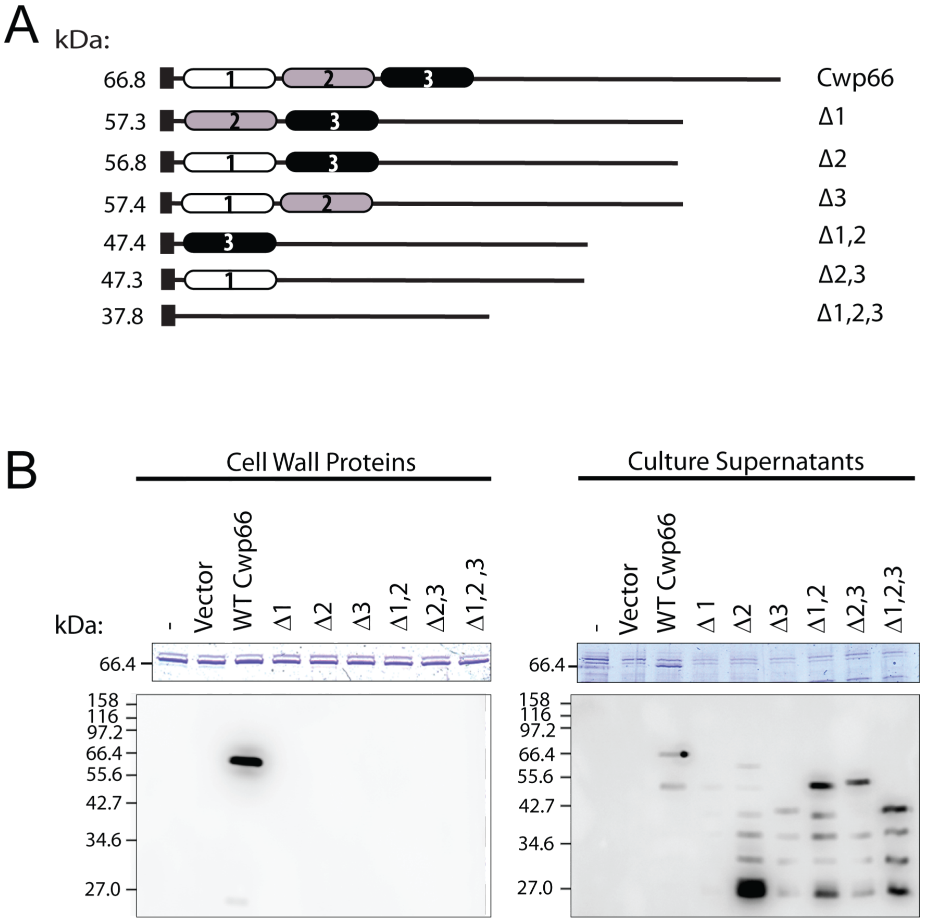

Fig. S1. Deletion of the CWB2 repeats in Cwp66 prevents cell wall attachment in *C. difficile*. A. Deletion derivatives of Cwp66 constructed. **B.** Cell wall extracts and culture supernatants from *C. difficile cwp66*::*erm* containing plasmids expressing the deletion derivatives of Cwp66 visualised by Coomassie blue staining (top) and western blotting using anti-Cwp66 antibody (bottom).

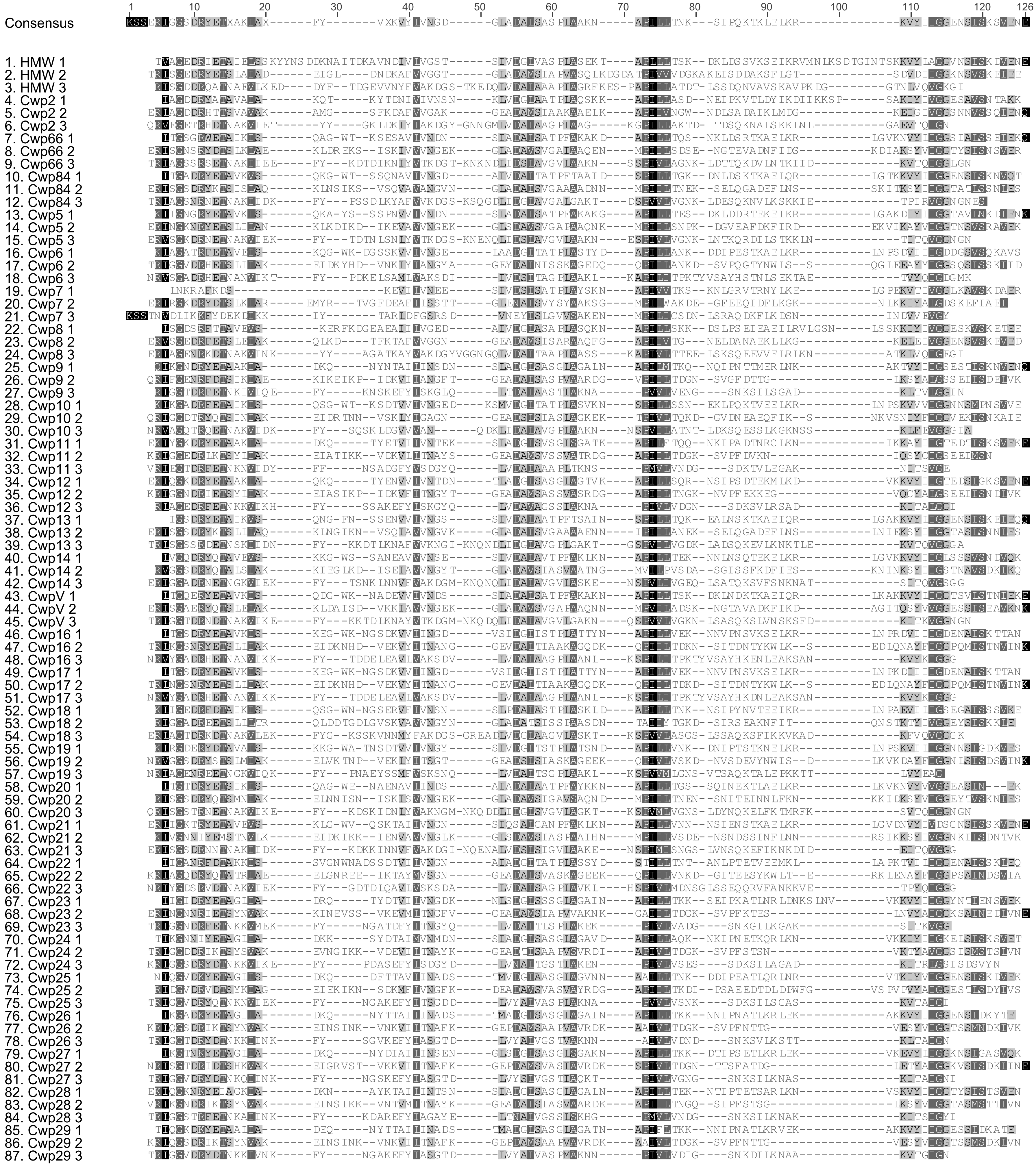
1
10
20
30
40
50
60
70
80
90
100
110
120
126
Consensus
1. HMW 1
2. HMW 2
3. HMW 3
4. Cwp2 1
5. Cwp2 2
6. Cwp2 3
7. Cwp66 1
8. Cwp66 2
9. Cwp66 3
10. Cwp84 1
11. Cwp84 2
12. Cwp84 3
13. Cwp5 1
14. Cwp5 2
15. Cwp5 3
16. Cwp6 1
17. Cwp6 2
18. Cwp6 3
19. Cwp7 1
20. Cwp7 2
21. Cwp7 3
22. Cwp8 1
23. Cwp8 2
24. Cwp8 3
25. Cwp9 1
26. Cwp9 2
27. Cwp9 3
28. Cwp10 1
29. Cwp10 2
30. Cwp10 3
31. Cwp11 1
32. Cwp11 2
33. Cwp11 3
34. Cwp12 1
35. Cwp12 2
36. Cwp12 3
37. Cwp13 1
38. Cwp13 2
39. Cwp13 3
40. Cwp14 1
41. Cwp14 2
42. Cwp14 3
43. CwpV 1
44. CwpV 2
45. CwpV 3
46. Cwp16 1
47. Cwp16 2
48. Cwp16 3
49. Cwp17 1
50. Cwp17 2
51. Cwp17 3
52. Cwp18 1
53. Cwp18 2
54. Cwp18 3
55. Cwp19 1
56. Cwp19 2
57. Cwp19 3
58. Cwp20 1
59. Cwp20 2
60. Cwp20 3
61. Cwp21 1
62. Cwp21 2
63. Cwp21 3
64. Cwp22 1
65. Cwp22 2
66. Cwp22 3
67. Cwp23 1
68. Cwp23 2
69. Cwp23 3
70. Cwp24 1
71. Cwp24 2
72. Cwp24 3
73. Cwp25 1
74. Cwp25 2
75. Cwp25 3
76. Cwp26 1
77. Cwp26 2
78. Cwp26 3
79. Cwp27 1
80. Cwp27 2
81. Cwp27 3
82. Cwp28 1
83. Cwp28 2
84. Cwp28 3
85. Cwp29 1
86. Cwp29 2
87. Cwp29 3

Fig. S2. Alignment of CWB2 motifs from *C. difficile*. The 87 CWB2 motifs from the 29 CWPs from *C. difficile* 630 were aligned using ClustalW and displayed using Geneious software. Highly conserved residues are shaded based on the Blosum 62 matrix; 100% similar, 80-100% similar, 60-80% similar and less than 60% similar. The motifs are numbered according to their location within their parent protein from N –> C terminus.

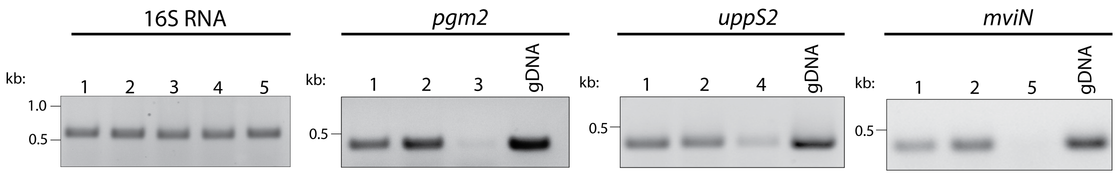

Fig. S3 Semi-quantitative RT-PCR of gene knock-down by expression of anti-sense RNA. Overnight cultures were diluted to an OD_{600} of 0.025 in BHIS broth. With the exception of 630, all cultures were supplemented with 500 ng/ml anhydrotetracycline. Cultures were grown to an OD_{600} of approximately 0.3 and cDNA prepared by reverse transcription of 1 µg of RNA. mRNA levels were then determined by semi-quantitative PCR. The strains are indicated as follows: 1 – 630; 2 – vector control, 3 – pSEW036 (*pgm2);* 4 - pSEW037 – (*uppS2);* 5 – pSEW038 (*mviN*). 25 cycles of PCR were used for amplification of 16S RNA, 35 cycles for amplification of *pgm2*, 32 cycles for amplification of *uppS2* and 28 cycles for amplification of *mviN.*

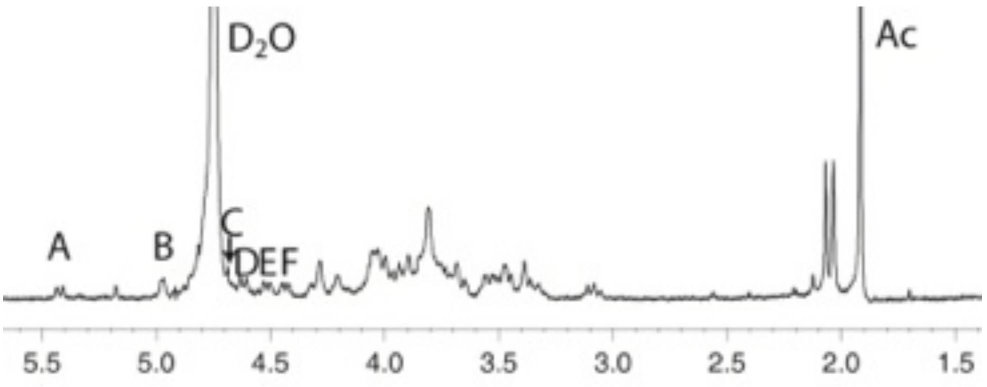

Fig. S4. ^{1}H NMR spectrum of the PS fraction purified from PG-PS. The ^{1}H NMR spectrum profile is similar to that of PSII (Ganeshapillai *et al* 2008). No other singlet, except the singlet between 1.9 and 2 which corresponds to acid acetic (Ac), was detected. The six anomeric resonances (designated A-F) and the two singlet resonances between 2.0 and 2.1 ppm were identified by Ganeshapillai *et al 2*008 as belonging to two D-Gal*p*NAc units.

Table S1 Plasmids created in this study

Plasmid	Relevant characteristics	Primers
pHAS002	pRFP144 based shuttle plasmid; expresses Cwp2	NF1304, NF1305
pZLS002	pRFP144 based shuttle plasmid; expresses Cwp66	NF1302, NF1303
pSEW002	Expression of Cwp2 lacking CWB_2 repeat 1	NF1552, NF1553
pSEW003	Expression of Cwp2 lacking CWB_2 repeat 2	NF1554, NF1555
pSEW004	Expression of Cwp2 lacking CWB_2 repeat 3	NF1556, NF1557
pSEW005	Expression of Cwp2 lacking CWB_2 repeat 1 and 2	NF1552, NF1555
pSEW006	Expression of Cwp2 lacking CWB_2 repeat 2 and 3	NF1557, NF1554
pSEW007	Expression of Cwp2 lacking CWB_2 repeat 1, 2 and 3	NF1557, NF1554
pSEW030	Expression of Cwp66 lacking CWB_2 repeat 1	NF1756, NF1757
pSEW031	Expression of Cwp66 lacking CWB_2 repeat 2	NF1758, NF1759
pSEW032	Expression of Cwp66 lacking CWB_2 repeat 3	NF1760, NF1761
pSEW040	Expression of Cwp66 lacking CWB_2 repeat 1 and 2	NF1756, NF1759
pSEW041	Expression of Cwp66 lacking CWB_2 repeat 2 and 3	NF1761, NF1758
pSEW042	Expression of Cwp66 lacking CWB_2 repeat 1, 2 and 3	NF1756, NF1761
pSEW056	Expression of Cwp66 with PILL residues of CWB_2 repeat 1mutated to AAAA	NF2163, NF2164
pSEW057	Expression of Cwp66 with PILL residues of CWB_2 repeat 2mutated to AAAA	NF2165, NF2166
pSEW058	Expression of Cwp66 with PILL residues of CWB_2 repeat 3mutated to AAAA	NF2167, NF2168
pSEW059	Expression of Cwp66 with PILL residues of CWB_2 repeat 1, 2 and3 mutated to AAAA	NF2163, NF2164, NF2165, NF2166, NF2167, NF2168
pSEW067	Expression of Cwp66 with PILL residues of CWB_2 repeat 1 and 2mutated to AAAA	NF2163, NF2164, NF2165, NF2166
pSEW068	Expression of Cwp66 with PILL residues of CWB_2 repeat 2 and 3mutated to AAAA	NF2165, NF2166, NF2167, NF2168
pSEW069	Expression of Cwp66 with PILL residues of CWB_2 repeat 1 and 3mutated to AAAA	NF2163, NF2164, NF2167, NF2168
pSEW085	Expression of Cwp66 with P of CWB_2 repeat 1 mutated to A	NF2664, NF2665

pSEW086	Expression of Cwp66 with P of CWB_2 repeat 2 mutated to A	NF2666, NF2667
pSEW087	Expression of Cwp66 with P of CWB_2 repeat 3 mutated to A	NF2668, NF2669
pSEW091	Expression of Cwp66 with P of CWB_2 repeat 1 and 2 mutated to A	NF2664, NF2665, NF2666, NF2667
pSEW092	Expression of Cwp66 with P of CWB_2 repeat 1 and 3 mutated to A	NF2664, NF2665, NF2668, NF2669
pSEW093	Expression of Cwp66 with P of CWB_2 repeat 2 and 3 mutated to A	NF2666, NF2667, NF2668, NF2669
pSEW096	Expression of Cwp66 with P of CWB_2 repeat 1, 2 and 3 mutated to A	NF2664, NF2665, NF2666, NF2667 NF2668, NF2669
pSEW079	Expression of Cwp2 with CWB_2 repeat 2 replaced with CWB_2repeat 1	NF2541, NF2542
pSEW080	Expression of Cwp2 with CWB_2 repeat 3 replaced with CWB_2 repeat 1	NF2543, NF2544
pSEW081	Expression of Cwp2 with CWB_2 repeat 1 replaced with CWB_2repeat 2	NF2545, NF2546
pSEW082	Expression of Cwp2 with CWB_2 repeat 3 replaced with CWB_2repeat 2	NF2547, NF2548
pHAS003	Retargeted vector for construction of *erm::cwp2* mutant	
pZLS003	Retargeted vector for construction of *erm::cwp66* mutant	

Table S2 Oligonucleotides used in this study

Name	Sequence (5' to 3')	Description
NF1552	CAATTTAACCTTTGAATTACCTTCTAATG	To remove CWB_1 of Cwp2 by inverse PCR
NF1553	CAATTAGAATCAGTAACTAAGAATATTG	To remove CWB_1 of Cwp2 by inverse PCR
NF1554	AATATTCTTAGTTACTGATTCTAATTGC	To remove CWB_2 of Cwp2 by inverse PCR
NF1555	CTTGCCGATATTGATAAAGATAGAAAAG	To remove CWB_2 of Cwp2 by inverse PCR
NF1556	AACTTTTCTATCTTTATCAATATCGGC	To remove CWB_3 of Cwp2 by inverse PCR
NF1557	GGAGTTGAATTGACAGTAATACAAAAG	To remove CWB_3 of Cwp2 by inverse PCR
NF1756	TGTATCTTTTGTAGATGCATTAGATG	To remove CWB_1 of Cwp66 by inverse PCR
NF1757	TTAAATGCAGAAAATATAAATTTTG	To remove CWB_1 of Cwp66 by inverse PCR
NF1758	AAAATTTATATTTTCTGCATTTAATTG	To remove CWB_2 of Cwp66 by inverse PCR
NF1759	AGTTTACCAAATGCAACAAGAATAGC	To remove CWB_2 of Cwp66 by inverse PCR
NF1760	TGCATTTGGTAAACTTCTTTCTACAG	To remove CWB_3 of Cwp66 by inverse PCR
NF1761	AATGAAAATGTTGTAGAAGATATAC	To remove CWB_3 of Cwp66 by inverse PCR
NF2163	GCTGCTGCTGCTACTCAAAGTAATAAATTGGATAG	To mutate PILL residues of CWB_2 repeat 1 of Cwp66 to AAAA by inverse PCR
NF2164	CGCATCTTTTGCTTTAGCAAATGG	To mutate PILL residues of CWB_2 repeat 1 of Cwp66 to AAAA by inverse PCR
NF2165	GCTGCTGCTGCTTCTGATTCAGAGAATGG	To mutate PILL residues of CWB_2 repeat 2 of Cwp66 to AAAA by inverse PCR
NF2166	CATGTTTTCTTGAGCAGCTATAGCTCC	To mutate PILL residues of CWB_2 repeat 2 of Cwp66 to AAAA by inverse PCR
NF2167	GCTGCTGCTGCTGCAGGAAATAAGCTTGATAC	To mutate PILL residues of CWB_2 repeat 3of Cwp66 to AAAA by inverse PCR
NF2168	AGAACTATTTTTAGCTGCTAATAC	To mutate PILL residues of CWB_2 repeat 3 of Cwp66 to AAAA by inverse PCR
NF2664	GCTATATTATTAACTCAAAGTAATAAATTGGATAGTAG	To mutate P of PILL residues of CWB_2 repeat 1 of Cwp66 to AAAA by inverse PCR
NF2665	CGCATCTTTTGCTTTAGCAAATG	To mutate P of PILL residues of CWB_2 repeat 1 of Cwp66 to AAAA by inverse PCR
NF2666	GCTATAATACTTTCTGATTCAGAGAATGGAAC	To mutate P of PILL residues of CWB_2 repeat 2 of Cwp66 to AAAA by inverse PCR
NF2667	CATGTTTTCTTGAGCAGCTATAGCT	To mutate P of PILL residues of CWB_2 repeat 2 of Cwp66 to AAAA by inverse PCR
NF2668	GCTATAGTATTAGCAGGAAATAAGCTTG	To mutate P of PILL residues of CWB_2 repeat 3 of Cwp66 to AAAA by inverse PCR
NF2669	AGAACTATTTTTAGCTGCTAATACACC	To mutate P of PILL residues of CWB_2 repeat 3 of Cwp66 to AAAA by inverse PCR
NF2541	aatcagtaactaagaatattATAGCTGGAGATGATAGATATG	Replace CWB_2 repeat 2 with CWB_2 repeat 1 in Cwp2 using Gibson's assembly
NF2542	tttatcaatatcggcaagCTTTTTAGCTGTATTTGATACTGC	Replace CWB_2 repeat 2 with CWB_2 repeat 1 in Cwp2 using Gibson's assembly
NF2543	ttgataaagatagaaaagttATAGCTGGAGATGATAGATATG	Replace CWB_2 repeat 3 with CWB_2 repeat 1 in Cwp2 using Gibson's assembly
NF2544	actgtcaattcaactccCTTTTTAGCTGTATTTGATACTGC	Replace CWB_2 repeat 3 with CWB_2 repeat 1 in Cwp2 using Gibson's assembly
NF2545	gtaattcaaaggttaaattgGAAAGACTAGCTGGAGATG	Replace CWB_2 repeat 1 with CWB_2 repeat 2 in Cwp2 using Gibson's assembly
NF2546	tagttactgattctaattgTTGATTTTCAATTTGGCTAGATAC	Replace CWB_2 repeat 1 with CWB_2 repeat 2 in Cwp2 using Gibson's assembly
NF2547	ttgataaagatagaaaagttGAAAGACTAGCTGGAGATG	Replace CWB_2 repeat 3 with CWB_2 repeat 2 in Cwp2 using Gibson's assembly
NF2548	actgtcaattcaactccTTGATTTTCAATTTGGCTAGATAC	Replace CWB_2 repeat 3 with CWB_2 repeat 2 in Cwp2 using Gibson's assembly

NF2549	gtaattcaaaggttaaattgCAAAGAGTTGAAGGAGAAAC	Replace CWB_2 repeat 1 with CWB_2 repeat 2 in Cwp2 using Gibson's assembly
NF2550	tagttactgattctaattgATTACCTATTTGAGTTACTTCTGC	Replace CWB_2 repeat 1 with CWB_2 repeat 2 in Cwp2 using Gibson's assembly
NF2551	aatcagtaactaagaatattCAAAGAGTTGAAGGAGAAAC	Replace CWB_2 repeat2 with CWB_2 repeat 3 in Cwp2 using Gibson's assembly
NF2552	tttatcaatatcggcaagATTACCTATTTGAGTTACTTCTGC	Replace CWB_2 repeat2 with CWB_2 repeat 3 in Cwp2 using Gibson's assembly
NF1810	gatc ggatcc AATTACCTTGAAATTTTAAATTAG	To amplify DNA from 630 gDNA for antisense RNA knock down of CD630_2780 (BamHI)
NF1811	gatc gagctc CTCTAAATTTTTATAAAATC	To amplify DNA from 630 gDNA for antisense RNA knock down of CD630_2780 (SacI)
NF1812	gatc ggatcc GATAATAATACCTATTG	To amplify DNA from 630 for antisense RNA knock down of CD630_2762 (BamHI)
NF1813	gatc gagctc CTCTACATAATCTATAAG	To amplify DNA from 630 for antisense RNA knock down of CD630_2762 (SacI)
NF1841	gatc ggatcc AGTTCTACTTTTAGAGTTTAAATTAG	To amplify DNA from 630 for antisense RNA knock down of CD630_2781 (BamHI)
NF1842	gatc gagctc CATACCTGTCCCATAGAAATTTG	To amplify DNA from 630 for antisense RNA knock down of CD630_2781 (SacI)
NF2734	CTGTAACAAATTGCTCTGTAAATAAAAGAC	To determine mRNA levels of CD630_2781 by RT PCR
NF2735	CTAAAGCAGCATTGTGGATTATG	To determine mRNA levels of CD630_2781 by RT PCR
NF2736	TGCTTTTATTTGACCTTCTTTGGCTTTAATTC	To determine mRNA levels of CD630_2762 by RT PCR
NF2737	TAGAAGGTGGGCAAGTCAAAATGGTATGAC	To determine mRNA levels of CD630_2762 by RT PCR
NF2738	CAAAAATATAAGCCTTTACACCATAAGCAC	To determine mRNA levels of CD630_2780 by RT PCR
NF2739	GAACAAACGAAAAATGAGCTATTAAGTATAAAAG	To determine mRNA levels of CD630_2780 by RT PCR
NF408	TCTTGAATATCAAAGGTGAGCCAGTACA	To amplify 16 S RNA of C. difficile
NF409	TACAGCGTGGACTACCAGGGTATCTAAT	To amplify 16 S RNA of C. difficile
